# Treating hematologic immune dysregulation in inborn errors of immunity: a real-life multicenter study

**DOI:** 10.3389/fimmu.2026.1854044

**Published:** 2026-07-27

**Authors:** Giorgio Costagliola, Filippo Consonni, Riccardo Castagnoli, Mayla Sgrulletti, Eleonora Gambineri, Giuliana Giardino, Federica Cavone, Davide Montin, Francesca Conti, Baldassarre Martire, Matteo Chinello, Irene D’Alba, Francesco Saettini, Adele Civino, Gian Luigi Marseglia, Roberta Romano, Beatrice Rivalta, Fabiola Guerra, Emilia Cirillo, Lucia Pacillo, Francesca Cillo, Laura Grilli, Federico Diomeda, Mattia Moratti, Francesca Robasto, Caterina Cancrini, Gabriella Casazza, Claudio Pignata, Viviana Moschese, Rita Consolini

**Affiliations:** 1 Section of Pediatric Hematology and Oncology, Azienda Ospedaliero-Universitaria Pisana, Pisa, Italy; 2 Department of Experimental and Clinical Biomedical Sciences “Mario Serio,” University of Florence, Florence, Italy; 3 Department of Clinical and Experimental Sciences, University of Brescia, Brescia, Italy; 4 Pediatric Immunology Unit, Pediatrics Clinic, ASST Spedali Civili di Brescia, Brescia, Italy; 5 Pediatric Unit, Department of Clinical, Surgical, Diagnostic and Pediatric Sciences, University of Pavia, Pavia, Italy; 6 Pediatric Clinic, Fondazione IRCCS Policlinico San Matteo, Pavia, Italy; 7 Pediatric Immunopathology and Allergology Unit, Policlinico Tor Vergata, University of Rome Tor Vergata, Rome, Italy; 8 Division of Pediatric Oncology/Hematology, Meyer Children’s Hospital IRCCS, Florence, Italy; 9 Department of Neurosciences, Psychology, Drug Research and Child Health (NEUROFARBA), University of Florence, Florence, Italy; 10 Department of Translational Medical Sciences, Pediatric Section, University of Naples Federico II, Naples, Italy; 11 Division of Pediatric Immunology and Rheumatology, Department of Public Health and Pediatrics Sciences, Regina Margherita Children’s Hospital, University of Turin, Turin, Italy; 12 Pediatric Unit, IRCCS Azienda Ospedaliero-Universitaria di Bologna, Bologna, Italy; 13 Department of Medical and Surgical Sciences (DIMEC), University of Bologna, Bologna, Italy; 14 Pediatrics and Neonatology Unit, Maternal-Infant Department, “Monsignor A. R Dimiccoli” Hospital, Barletta, Italy; 15 Pediatric Hematology-Oncology, Department of Mother and Child, Azienda Ospedaliera Universitaria Integrata Verona, Verona, Italy; 16 Paediatric Haematology-Oncology, Maternal Infant Hospital “G. Salesi,”, Ancona, Italy; 17 Centro Tettamanti, Fondazione IRCCS San Gerardo Dei Tintori, Via Cadore, Monza, Italy; 18 Pediatria, Fondazione IRCCS San Gerardo dei Tintori, Monza, Italy; 19 Pediatric Rheumatology and Immunology, “Vito Fazzi” Hospital, Lecce, Italy; 20 Research Unit of Primary Immunodeficiencies, Unit of Clinical Immunology and Vaccinology, IRCCS Bambino Gesù Children Hospital, Rome, Italy; 21 Dipartimento Di Medicina E Chirurgia, Università Degli Studi Milano-Bicocca, Milan, Italy; 22 Specialty School of Paediatrics - University of Bologna, Bologna, Italy; 23 Department of Biomedicine and Prevention, PhD in Immunology, Molecular Medicine and Applied Biotechnology, University of Rome Tor Vergata, Rome, Italy; 24 Department of Systems Medicine, University of Rome Tor Vergata, Rome, Italy; 25 Section of Clinical and Laboratory Immunology, University of Pisa, Pisa, Italy

**Keywords:** abatacept, autoimmune lymphoproliferative disease, HSCT, JAK inhibitors, leniolisib, sirolimus

## Abstract

**Background:**

Hematologic immune dysregulation (H-ID) – including autoimmune cytopenia (AIC), lymphoproliferation (LPD), and hemophagocytic lymphohistiocytosis (HLH) – is a potentially life-threatening manifestation of inborn errors of immunity (IEI). Despite the availability of targeted therapies, its management remains challenging, with no standardized treatment strategies established.

**Objective:**

To evaluate the efficacy and safety of the available treatments for H-ID, with a specific focus on the treatment indications and outcome differences between targeted versus non-targeted therapies.

**Methods:**

This multicentric retrospective study included 116 patients with IEI and H-ID across Italian tertiary care centers. Data included treatment indications, response rate, and incidence of adverse events (AEs).

**Results:**

We included patients with autoimmune lymphoproliferative immunodeficiencies (ALPID, 37.1%), humoral immunodeficiencies (31%), syndromic IEI (8.6%), and combined immunodeficiencies (8.6%). H-ID consisted of AIC (67.2%), LPD (71.6%), and HLH (9.5%). 85.3% of patients received treatment, including on-demand therapies (37.9%), long-term treatments (LTT, 84.8%), and hematopoietic stem-cell transplantation (HSCT; 9.1%). LTT included sirolimus (42.9%), mycophenolate mofetil (34.5%), rituximab (29.8%), and targeted therapies (17.9%). Sirolimus showed a higher complete response (CR) rate (61.1%), especially in ALPID patients and those with lymphoproliferation (72%). CR rate was significantly higher in patients receiving targeted therapies (80% vs 43.4%, *p* < 0.05). AEs were reported in 13.3% of cases, leading to drug withdrawal in 3.6%.

**Conclusions:**

The indications for LTT and HSCT in H-ID are still heterogeneous. Response rates are variable and influenced by the underlying diagnosis. Targeted therapies are associated with an improved response, the suboptimal molecular diagnosis rate currently limits their use.

## 1 Background

In recent years, immune dysregulation has been increasingly recognized as a presenting or leading feature of inborn errors of immunity (IEI). Indeed, immune dysregulation is part of the clinical phenotype of a wide range of IEI and depends on different, and significantly overlapped, molecular mechanisms (1, 2). The most common manifestations of immune dysregulation are lymphoproliferation (LPD) and autoimmunity, with autoimmune cytopenia (AIC) being the most frequently reported autoimmune feature. LPD is described in up to 40% of patients of the European Society for Immunodeficiencies (ESID) registry (3), while the frequency of AIC is reported to be 120-fold higher in IEI when compared to the general population (4, 5). Also, hemophagocytic lymphohistiocytosis (HLH) can be part of the clinical spectrum of several IEIs (6). Collectively, LPD, AIC, and HLH represent the main components of the phenotypic spectrum of hematologic immune dysregulation (H-ID).

Several studies have addressed therapeutic strategies that directly target the underlying molecular defects in selected IEI with immune dysregulation. These options include JAK inhibitors for JAK/STAT-related disorders (e.g., STAT1 and STAT3 gain of function [GOF]), abatacept for CTLA-4 haploinsufficiency and LRBA deficiency, and leniolisib for the activated phosphoinositide 3-kinase delta syndrome (APDS) (7–11). Additionally, for a few monogenic conditions, it is possible to adopt therapies that target disease-specific overactivated molecular pathways. This is the case of sirolimus for autoimmune lymphoproliferative syndrome (ALPS) caused by *FAS* mutations (ALPS-FAS, ALPS-somatic FAS, or ALPS-sFAS) and for APDS (12, 13), and for the use of etanercept in patients with deficiency of adenosine deaminase 2 (DADA2) (14). Together, targeted therapeutic approaches opened new opportunities for the treatment of H-ID. However, a molecular diagnosis is available only for a subset of patients presenting with H-ID, thus significantly limiting a broader adoption of targeted therapies in clinical practice. Therefore, most patients are treated with immunomodulatory agents based on a phenotypic-directed approach and, in selected cases, undergo hematopoietic stem cell transplantation (HSCT) (15).

Given the heterogeneity of IEI and the variable clinical presentation and combinations of dysregulatory features, there is no uniform consensus on the optimal therapeutic approach in this subset of patients. In this study, we aimed to analyze the response rates and occurrence of adverse events (AEs) associated with individual therapeutic agents used in H-ID, with a specific focus on comparing targeted versus non-targeted (phenotypic-directed) therapies.

## 2 Methods

### 2.1 Patient selection

A retrospective multicentric study was performed on patients with IEI followed in centers of the Italian Pediatric Research Society from 2014 to 2024, in whom H-ID (AIC, LPD, HLH) represented the leading disease sign and the primary target of medical treatment.

All the included patients had a diagnosis of IEI formulated according to genetic features or current ESID diagnostic criteria (16). Genetic analysis was performed according to the clinical practice standards of each center, with most of patients undergoing IEI-specific next-generation sequencing (NGS) or whole exome sequencing (WES) panels. In specific cases and based on the clinical suspicion, other investigations were performed, including single-gene (Sanger) sequencing, CGH-array, analysis of somatic mutations, or whole genome sequencing.

Patients were classified into subgroups according to their clinical features and molecular diagnosis. These included humoral IEI, IEI with syndromes, autoimmune lymphoproliferative immunodeficiencies (ALPID), combined immunodeficiencies (CID), and autoinflammatory disorders. The classification of patients into the ALPID subgroup was made according to the diagnostic criteria published by Magerus et al. (17).

### 2.2 Data collection

Data collection included the clinical and molecular diagnosis, disease phenotype (most relevant infectious and immune dysregulation features), and treatment strategies. Treatment response was categorized as complete response (CR), partial response (PR), or no response (NR). The response to chemotherapy in patients with clonal LPD was not evaluated, as this analysis falls outside the primary objectives of this study.

### 2.3 Outcome measures

Treatment response for immune thrombocytopenia (ITP), autoimmune hemolytic anemia (AHA), and autoimmune neutropenia (AIN) was defined according to previously established standards (18–20). The response to HLH-specific protocols (i.e. HLH 2004 protocol) (21) was assessed according to previously published response criteria (22, 23). For LPD, treatment response was quantified through clinical and radiological assessment (**Supplementary Table 1**). When patients presented multiple features of H-ID, the overall response was determined by the combination of the response rate observed for the different clinical features, as detailed in **Supplementary Table 1**.

Adverse events were classified according to the Common Terminology Criteria for Adverse Events (CTCAE) grading system.

### 2.4 Statistical analysis

Statistical analyses were performed using GraphPad Prism v10.0 (GraphPad Software, San Diego, CA). Normality of continuous variables was assessed. Data are reported as medians (range). Group comparisons used Student’s t-test or Mann–Whitney U test, as appropriate. Categorical data were expressed as counts and percentages and analyzed with chi-square or Fisher’s exact test. A *p-value* < 0.05 was considered statistically significant.

### 2.5 Ethics approval

This study was conducted in accordance with the Declaration of Helsinki. The participants or their guardians provided written informed consent for the publication of any data included in this article. The study was approved by the local ethics committee of each participating center, and coordinating ethics approval was granted by the Regional Pediatric Ethics Committee of Tuscany, based at Meyer Children’s Hospital IRCCS (Florence, Italy; ORG-ID 100032435).

## 3 Results

### 3.1 Demographic features

A total of 152 patients with IEI and immune dysregulation were initially evaluated for study eligibility; 36 were excluded due to the absence of a prominent hematological involvement.

Consequently, a cohort of 116 patients with H-ID (63 males, 53 females) was enrolled. The mean age at H-ID onset was 9.1 ± 8.9 years, while the mean age at clinical or molecular diagnosis was 12.7 ± 12.9 years. H-ID was present at disease onset in 76.7% of patients. The median follow-up was 5 years.

A molecular diagnosis was available for 64 patients (55.2%) (**Table 1**, detailed in **Supplementary Table 2**). The most frequent disease categories were autoimmune lymphoproliferative immunodeficiency (ALPID; n: 43; 37.1%), common variable immunodeficiency (CVID) and other humoral immunodeficiencies (n: 36; 31%), combined immunodeficiencies (CID; n:10; 8.6%), and IEIs with syndromes (n:10; 8.6%). In the ALPID group, 23/43 (53.5%) received a molecular diagnosis, and ALPS or ALPS-sFAS was diagnosed in 18.2% of patients (8/43). Of note, one patient was retained in the ALPS-CASP10 category, although the role of CASP10 variants in inducing defective FAS-mediated apoptosis and ALPS pathogenesis remains controversial (24). A molecular diagnosis was available in 12/36 patients (33.3%) with humoral immunodeficiency and 4/10 (40%) of those with CID. Twenty-six patients of this cohort were previously published in case reports or disease-specific cohort studies (24–44).

**Table 1:** Clinical and molecular diagnosis of enrolled patients.

Disease	Number	Molecular diagnosis	List
ALPID	43	23	APDS-1 (4) APDS-2 (1) ALPS-FAS (6); ALPS-sFAS (2), ALPS-CASP10 (1), CTLA4 haploinsufficiency (5), STAT3 GOF (2), STAT1 GOF (1), PKCD (1)
Other immune dysregulation disorders	9	8	XLP-1 (2); XLP-2 (2); fHLH-UNC13D (2); APECED (1); BACH2 deficiency (1);
CVID and other humoral immunodeficiencies	36	12	NFKB1 haploinsufficiency (3), NFKB2 haploinsufficiency (1), heterozygous TACI deficiency (5); IKAROS haploinsufficiency (1); IRF2BP2 deficiency (2)
Syndromic IEI	10	10	22q11.2DS (10)
Combined immunodeficiencies	10	4	Hypomorphic RAG 1 mutation (2), DCLRE1C mutation (1), BENTA syndrome (1)
Autoinflammatory disorders	4	4	DADA2 (2); NLRP12-associated disease (2)
Others	4	4	BCL11B-related disorder (1); Kabuki syndrome (3)

22q11.2DS, 22q11.2 deletion syndrome; ALPID, autoimmune lymphoproliferative immunodeficiencies; ALPS, autoimmune lymphoproliferative syndrome; APDS, activated phosphoinositide 3-kinase delta syndrome; APECED, Autoimmune polyendocrinopathy-candidiasis-ectodermal dystrophy; BENTA, B-cell Expansion with NF-kappaB and T-cell Anergy; DADA2, deficiency of adenosine deaminase 2; GOF, gain of function; PKCD, Protein kinase C δ deficiency; XLP-1, X-linked lymphoproliferative disease type 1, XLP-2, X-linked lymphoproliferative disease type 2.

### 3.2 Clinical features

**Figure 1:**
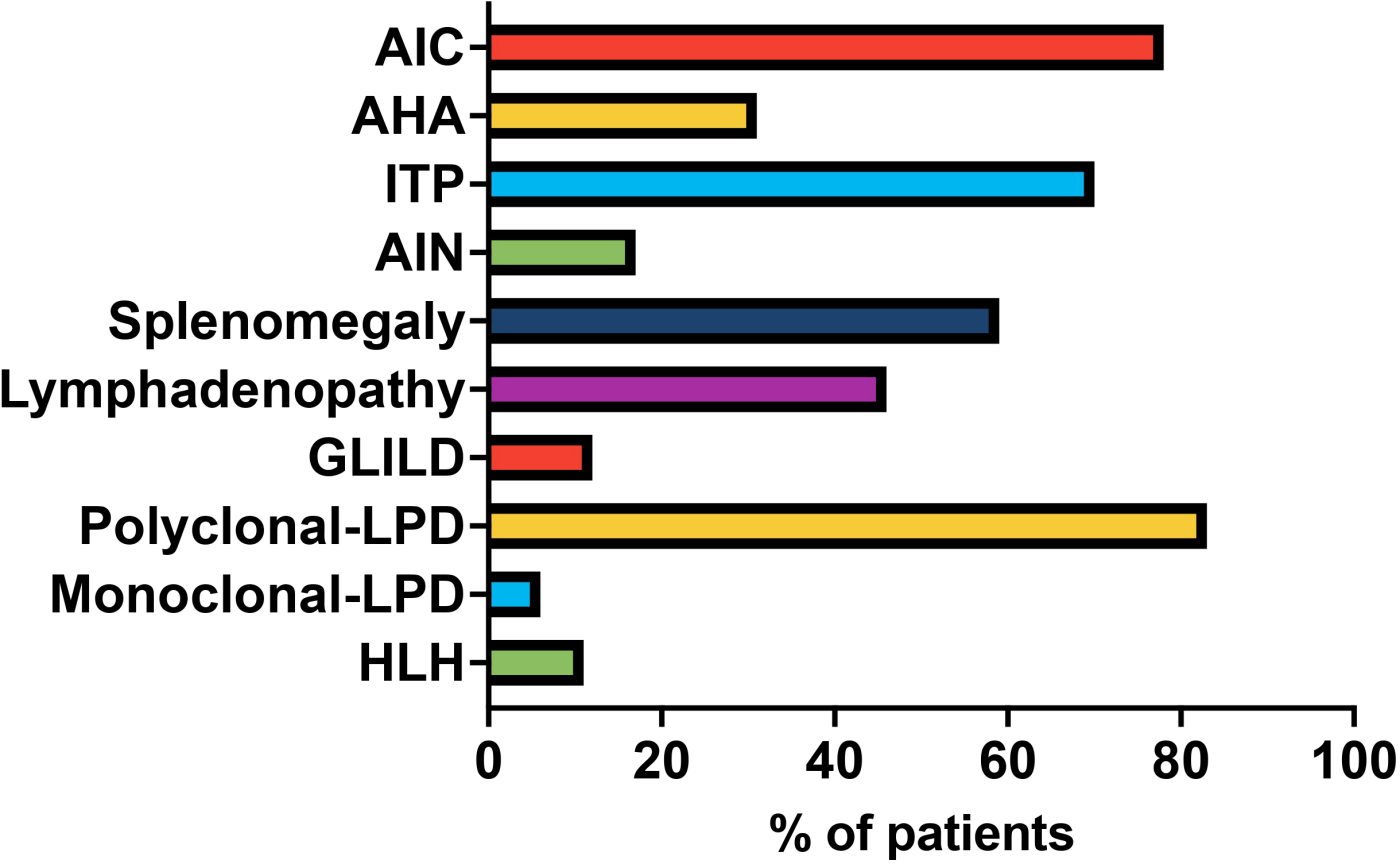
Overview of the clinical features of hematologic immune dysregulation. AHA, autoimmune hemolytic anemia; AIC, autoimmune cytopenia; AIN, autoimmune neutropenia; GLILD, granulomatous-lymphocytic interstitial lung disease; HLH, hemophagocytic lymphohistiocytosis; ITP, immune thrombocytopenia; LPD, lymphoproliferative disease.

H-ID consisted of LPD (n: 83; 71.6%), AIC (n: 78; 67.2%), and HLH (n: 11; 9.5%). Patients with cytopenia had ITP (n: 70/78; 89.7%), AHA (n: 31/78; 39.7%), and AIN (n: 17/78; 21.8%); multilineage cytopenia was present in 30/78 patients (38.5%). Among patients with LPD, polyclonal lymphoproliferative disease (pLPD) included recurrent/chronic lymphadenopathy (n: 46/83; 55.4%), splenomegaly (n: 59/83; 71.1%), or tissue infiltration as lymphocytic enteropathy or granulomatous lymphocytic interstitial lung disease (GLILD; n: 12/83; 14.5%); clonal LPD occurred in 6/83 patients (7.2%) (**Figure 1**). The association between multiple features of H-ID was reported in 25/43 patients in the ALPID group (%), 18/36 in the humoral IEI group (50%), 3/10 of the CID group (30%), and 5/10 (50%) in the group of syndromic IEIs.

Patients showed additional manifestations of immune dysregulation (**Supplementary Table 3**), which included atopy (n: 19; 16.4%), rheumatic diseases (n: 18; 15.5%), gastrointestinal disease (n: 18; 15.5%), and endocrinopathy (n: 15; 12.9%). Recurrent or severe infections have been reported in 58 patients (50%).

### 3.3 Treatment strategies

**Figure 2:**
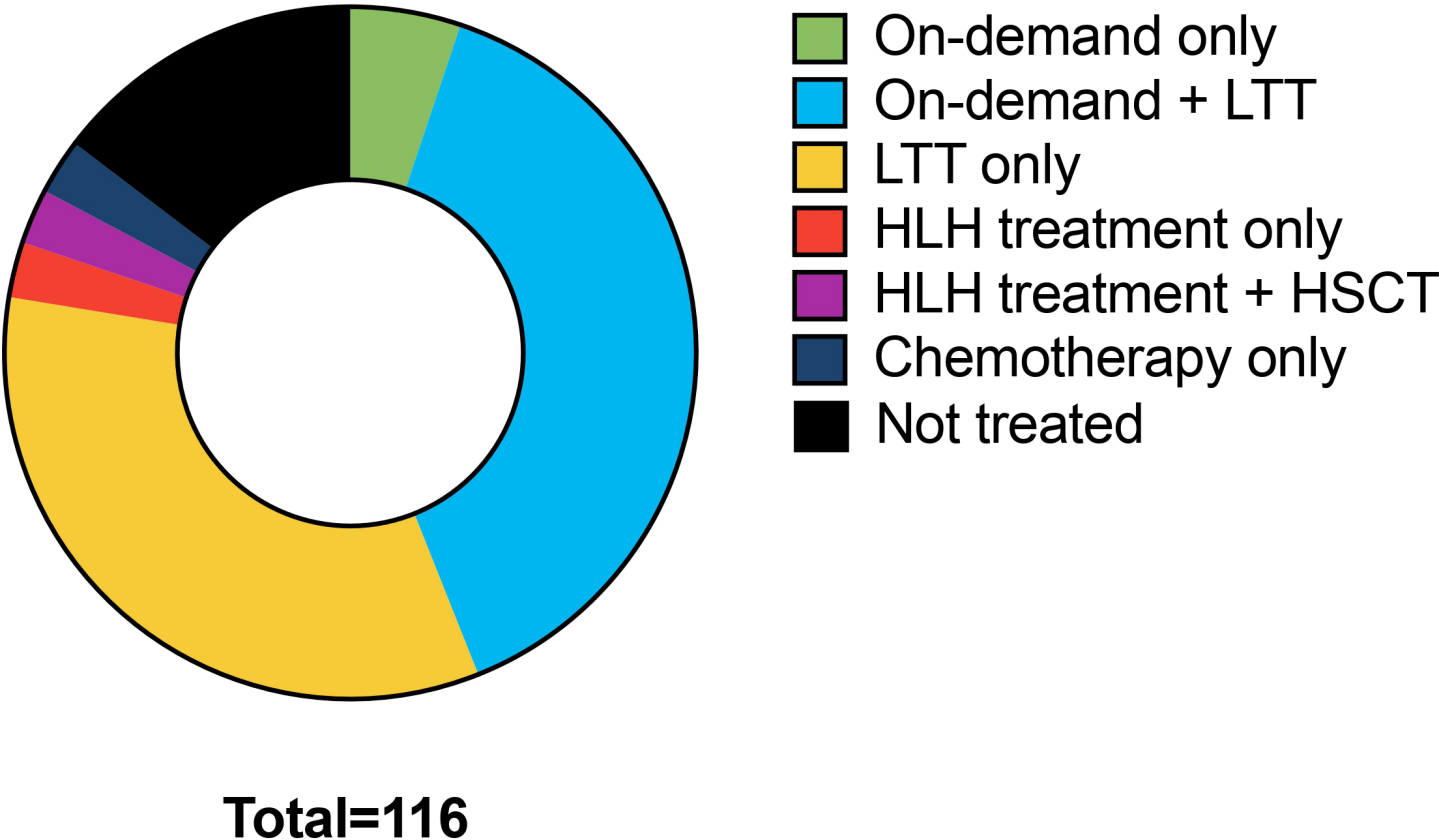
Overview of the treatment strategies for hematologic immune dysregulation in the analyzed cohort. HLH, hemophagocytic lymphohistiocytosis; HSCT, hematopoietic stem cell transplantation; LTT, long-term treatment.

Overall, 99 patients (85.3%) received treatment for H-ID. Among treated patients, 51 (51.5%) received on-demand treatments for AIC, and 84 (84.8%) received long-term treatment (LTT); 6 (6%) received only HLH treatment, which was followed by HSCT in 50% of cases (**Figure 2**), while 3 patients received only chemotherapy for clonal LPD. HSCT was performed in 9 patients (7.5%), while 2 patients (1.7%) underwent splenectomy for refractory AIC.

Among patients receiving LTT, 29/84 (34.5%) required at least two therapeutic lines (≥3 lines: n: 12; 14.3%). Among patients requiring at least two therapeutic lines, 19/29 (65.5%) had multiple features of H-ID with an association of AIC and pLPD, and 18/29 (62.1%) had a confirmed molecular diagnosis, which included 22q11.2 DS (3), TACI heterozygous mutation (3), diseases of the ALPID spectrum (STAT3 GOF:1; CTLA4 haploinsufficiency:1), and others.

The most commonly used agents for long-term disease management were sirolimus (n: 36/84; 42.9% of patients receiving LTT), mycophenolate mofetil (MMF; n: 29/84; 34.5%), and rituximab (n: 25/84; 29.8%). Additional conventional immunomodulators were administered for the indication of H-ID in 8/84 patients (9.5%) and included long-term corticosteroids (1%), cyclosporine (3.6%), azathioprine (2.4%), and methotrexate (2.4%). Thrombopoietin-receptor agonists (eltrombopag) were administered in 10/84 (11.9%) for ITP.

Targeted therapies were administered in 15 patients (17.9%) with a confirmed molecular diagnosis, and included sirolimus (n: 7/84; 8.3%) for ALPS or APDS, abatacept in CTLA-4 haploinsufficiency (n: 4/84; 4.8%), etanercept in DADA2 (n: 2/84; 2.4%), leniolisib in APDS (n: 1/84; 1.2%), and ruxolitinib in STAT1 GOF (n: 1/84; 1.2%).

Other monoclonal treatments, used according to a phenotypic-directed strategy, were used in 9/84 patients (10.7%) and consisted of bortezomib (n:1; 1.2%), eculizumab (n: 1; 1.2%), anakinra (6/84; 7.1%), and canakinumab (n: 1;1.2%).

Patients who did not receive treatment for H-ID had a clinical expression of isolated lymphoproliferation (n: 11/17; 64.7%), autoimmune neutropenia and lymphoproliferation (n: 3/17; 17.6%), or mild-to-moderate immune thrombocytopenia (n: 3/17; 17.6%).

Patients included in this cohort received also additional treatments not directed to H-ID (**Supplementary Table 4**).

### 3.4 Efficacy of on-demand treatment

On-demand treatments, administered to control AIC, included intravenous immunoglobulin (IVIG) and corticosteroids, each administered in 40 patients (40.4% of treated patients). Among patients treated with IVIG, CR was achieved in 20%, PR in 47.5%, while 32.5% of patients were classified as NR. Treatment with corticosteroids resulted in CR in 42.5% of patients, PR in 30%, and NR in 27.5%. Overall, the rate of NR was 30%.

### 3.5 Indications for treatment with conventional immunosuppressive/immunomodulating agents

**Figure 3:**
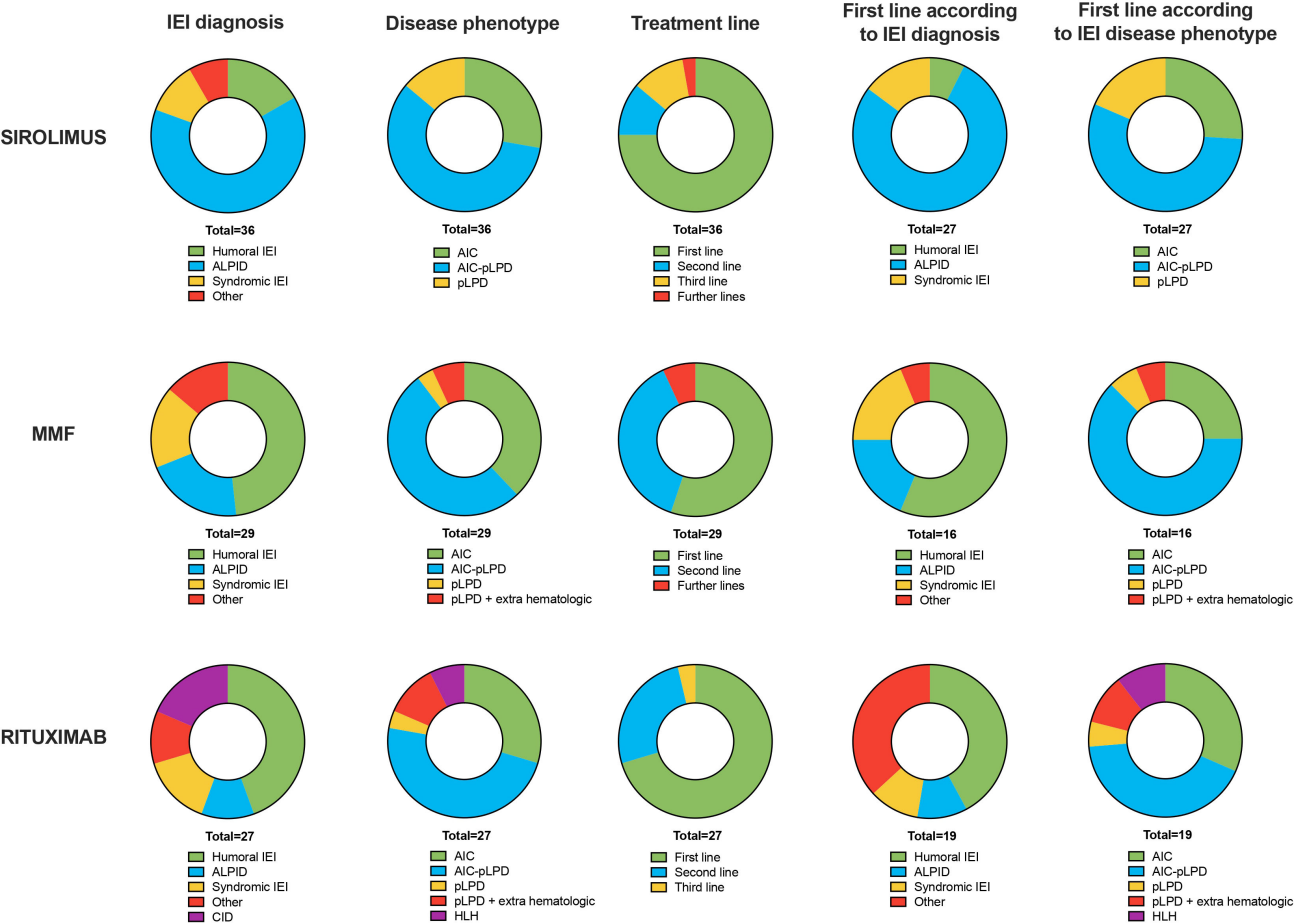
Therapeutic indications for conventional long-term treatments according to IEI diagnosis and disease phenotype. This figure illustrates, for each therapeutic agent (sirolimus, MMF, rituximab), the rate of administration according to the IEI diagnosis and clinical phenotype. Also, the rate of administration as a first-line therapy according to disease diagnosis and phenotype is presented. AIC, autoimmune cytopenia; ALPID, autoimmune lymphoproliferative immunodeficiencies; CID, combined immunodeficiencies; HLH, hemophagocytic lymphohistiocytosis; MMF, mycophenolate mofetil; LPD, lymphoproliferative disease.

Sirolimus was predominantly adopted in patients with ALPID, while MMF and rituximab were more frequently administered in patients with humoral IEI (**Figure 3**). In 19.4% of cases, sirolimus was used as target therapy, as further discussed.

Most patients were treated for AIC alone or in combination with pLPD, while only 10.7% of patients (9/84) were treated for isolated pLPD. In this case, sirolimus was the most used drug (55.5%). It is relevant to highlight that, in 6.5% of patients treated with MMF and 12% of those treated with rituximab, although H-ID consisted of isolated pLPD, the clinical picture underlying the therapeutic choice was broader (nephrotic syndrome: 60%, enteropathy: 40%).

### 3.6 Efficacy and safety of long-term treatments for AIC and LPD

**Figure 4:**
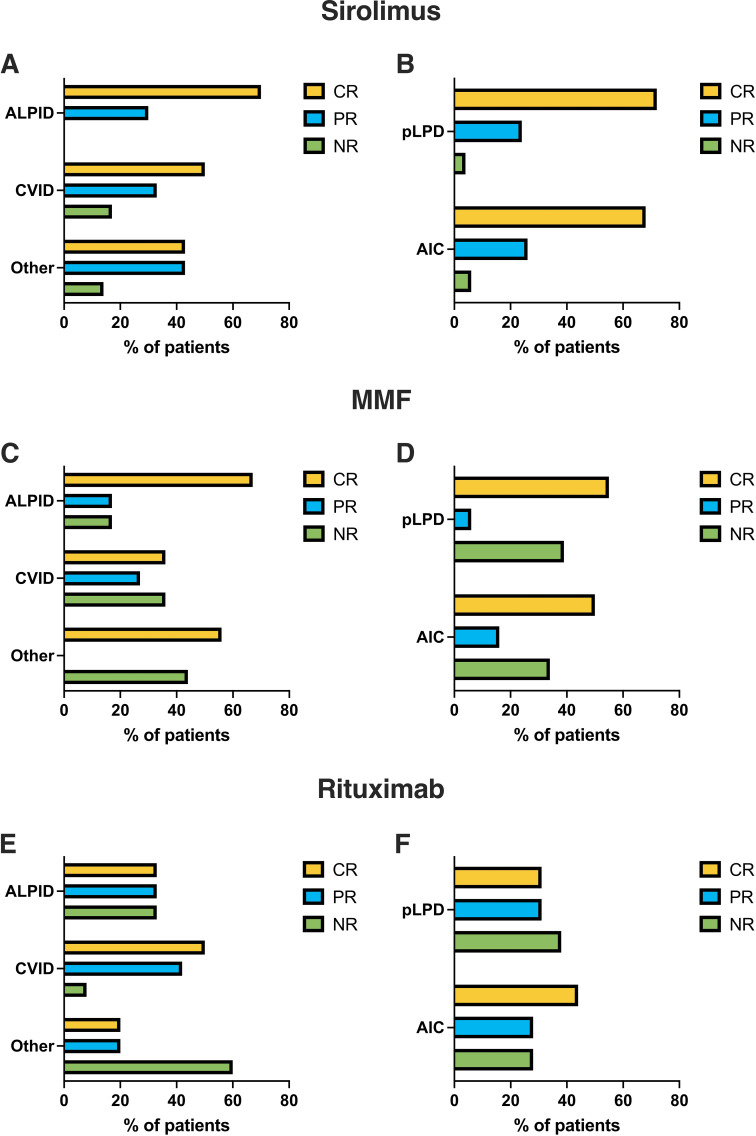
Treatment response to sirolimus, mycophenolate mofetil, and rituximab according to clinical diagnosis and disease phenotype. This figure illustrates the response rate (CR, PR, NR) to sirolimus **(A, B)**, MMF **(C, D)**, and rituximab **(E, F)**, according to the disease diagnosis (ALPID, CVID, others) and the clinical manifestations of H-ID (AIC, pLPD). AIC, autoimmune cytopenia; ALPID, autoimmune lymphoproliferative immunodeficiencies; CR, complete response; CVID, common variable immunodeficiency; MMF, mycophenolate mofetil; p-LPD, polyclonal lymphoproliferative disease; PR, partial response; NR, no response.

The efficacy and safety of the most employed conventional treatments are summarized in **Table 2**. Overall, patients treated with sirolimus, MMF, or rituximab showed a rate of CR of 50% and a rate of PR of 27.8%. **Figure 4** summarizes the different response rates stratified by clinical diagnosis and H-ID manifestations (AIC, pLPD).

**Table 2:** Efficacy and safety of long-term treatments for hematologic immune dysregulation.

Drug	N	CR	PR	NR	Adverse events	Description	Grade	Withdrawn for AE
Sirolimus	36	22 (61.1%)	12 (33.3%)	2 (5.6%)	9	Increased infectious rate (4)	2 (4 patients)	1 (ecthyma)
						Transaminitis (1)	2	
						Oral aphthosis (3)	1 (2 patients); 2 (2 patient)	
						Ecthyma (1)	3	
Mycophenolate mofetil	29	14 (48.3%)	5 (17.2%)	10 (34.5%)	3	Lymphopenia (1)	2	/
						Diarrhea (1)	2	
						Increased infectious rate (1)	2	
Rituximab	25*	9 (36%)	8 (32%)	8 (32%)	1	Increased infectious rate (1)	2	1
Eltrombopag	10	2 (20%)	6 (60)	2 (20%)	/			
Others	13	5 (38.5%)	1 (7.8%)	7 (53.8%)	3	Pneumonia (1)-MTX	3	1 (severe sepsis in a patient receiving cyclosporine)
						Severe sepsis (1)-cyclosporine	4	
Total	113	52 (46%)	32 (28.3%)	29 (25.7%)	15 (13.3%)			3 (2.6%)

Adverse events were classified according to the Common Terminology Criteria for Adverse Events (CTCAE) grading system.

*In 3 cases, rituximab was used for the treatment of HLH. Data are reported in **Supplementary Table 4**. AEs: adverse events.

In this cohort, AEs were reported in 16/99 patients (16%). Treatment discontinuation was necessary in 3/84 patients receiving LTT (3.6%) and depended on the occurrence of severe infections, requiring hospitalization (n: 2) or increased frequency of infections (n: 1).

### 3.7 Efficacy and safety of targeted therapies

The efficacy and safety of targeted therapies is summarized in **Table 3**. Except for one patient with DADA2 treated with etanercept, CR or PR was achieved by all patients.

**Table 3:** Efficacy and safety of targeted therapies.

Drug	N	Disease	Indication	Previous treatments	CR	PR	NR	Adverse events
Abatacept	4	CTLA-4 haploinsufficiency	AIC, pLPD (3); pLPD and systemic features (1)	High-dose steroids (3), azathioprine 1), MMF (1), eltrombopag (1), sirolimus (1), rituximab (1)	3	1		
Etanercept	2	DADA2	AIC, pLPD, systemic features (2)	Cyclosporine (1), methotrexate (1)	1		1	1 (pulmonary fungal disease)-grade 4
Leniolisib	1	APDS	LPD (polyclonal, clonal)	Chemotherapy+Ibrutinib+Brentuximab-bendamustina	1			
Ruxolitinib	1	STAT1 GOF	Secondary HLH prevention	HLH treatment	1			
Sirolimus	5	ALPS-FAS or ALPS-sFAS	pLPD+AIC (4); pLPD (1)	/	5			1 (pneumonia)-grade 2
	2	APDS	pLPD (1), pLPD+AIC (1)	/	1	1		1 (Pneumonia)-grade 3
Total	15				12 (80%)	2 (13.3%)	1 (6.7%)	3

Adverse events were classified according to the Common Terminology Criteria for Adverse Events (CTCAE) grading system.

AIC, autoimmune cytopenia; ALPS, autoimmune lymphoproliferative syndrome; APDS, activated phosphoinositide 3-kinase delta syndrome; LPD, lymphoproliferative disease; pLPD, polyclonal lymphoproliferative disease.

**Figure 5:**
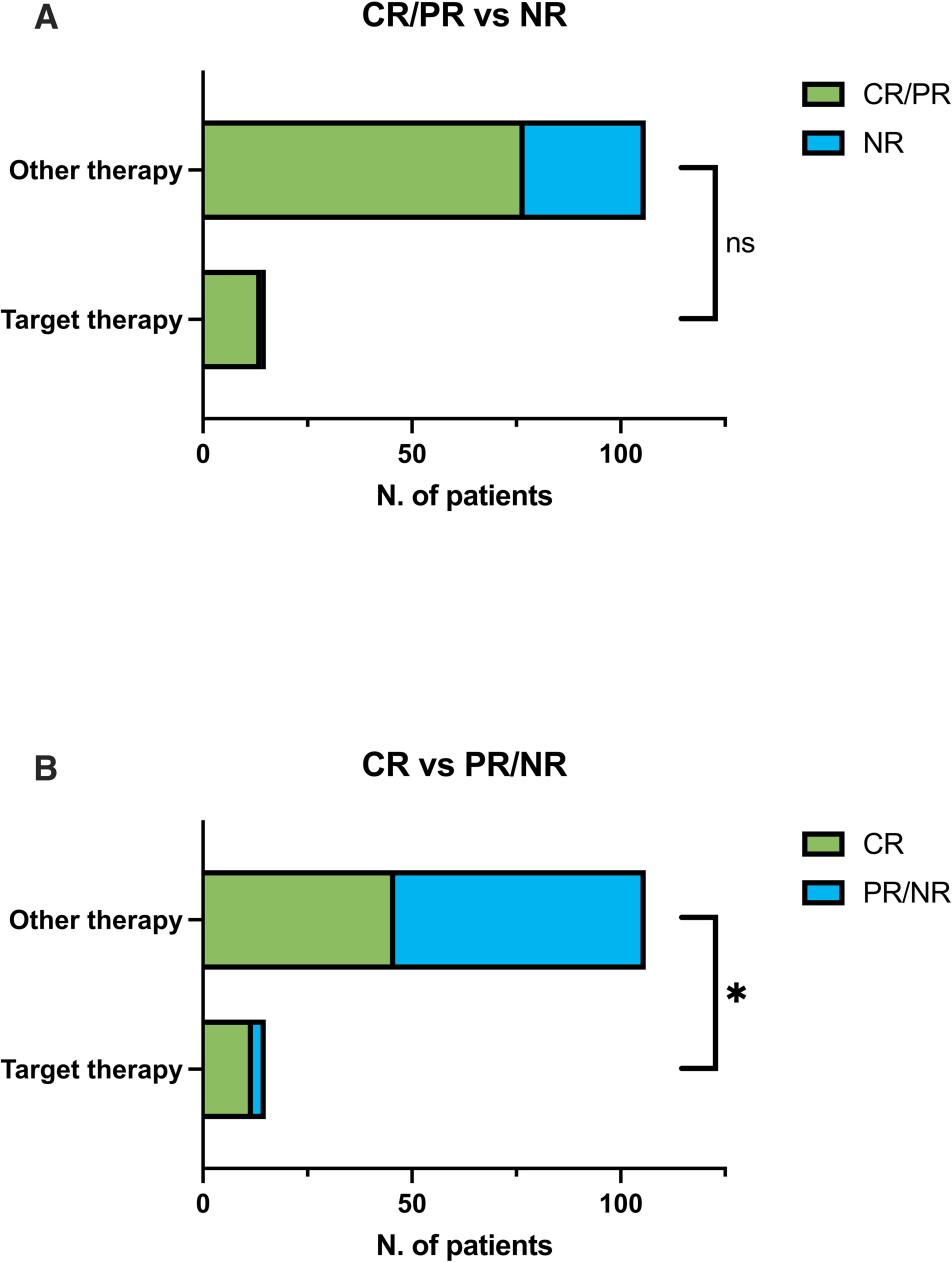
Treatment response in patients receiving targeted therapies versus other drugs. Overall response rate (CR + PR) to targeted therapies, compared to the rest of the cohort, was higher, although this difference was not statistically significant **(A)**. The rate of CR was significantly greater in the targeted therapy group **(B)**. CR, complete response; PR, partial response; NR, no response.

Patients receiving targeted therapies showed a higher overall response rate (CR + PR) compared to the rest of the cohort (93.3% vs. 72.6%), although this difference was not statistically significant. Notably, the rate of CR was significantly greater in the targeted therapy group (80% vs. 43.4%; p = 0.01)(**Figure 5**).

In this subgroup, AEs were reported in 3/15 patients (20%). The incidence of AEs was slightly higher among patients receiving targeted therapies compared to the rest of the cohort (20% vs. 12.3%), although this difference was not statistically significant. Notably, treatment discontinuation was required in only one case.

### 3.8 Response to HLH treatment

Treatment according to HLH-specific protocols was administered in 45.5% of patients with HLH, resulting in CR in 20%, PR in 40%, and NR in 40% (**Supplementary Table 5**). Additional drugs included anakinra (54.5%), rituximab (27.3%), ruxolitinib (18.2%), and canakinumab (9.1%). The overall HLH-related mortality was 27.3%.

### 3.9 Patients undergoing HSCT

The indications for HSCT included the underlying molecular diagnosis (33.3%), the refractoriness to H-ID treatments (n: 3; 33.3%), and HLH (n: 3; 33.3%). HSCT was preceded by different treatments, which included HLH-specific protocol (n: 3; 33.3%), conventional immunosuppressive/immunomodulating agents (n: 5; 55.5%), and targeted therapies (n: 2; 22.2%). HSCT was successful in 8/9 patients (median follow-up: 5 years), while one (diagnosed with fHLH) died of early complications (**Table 4**).

**Table 4:** Indications, conditioning regimens, and outcomes of patients undergoing hematopoietic stem cell transplantation.

Underlying disease	Age at HSCT (years)	Indication for HSCT	GvHD	Other HSCT-associated complications	Previous treatment	Outcome	Follow-up (years)
STAT3 GOF	8.5	Molecular diagnosis	Acute cutaneous and intestinal GvHD	VOD PRES	Sirolimus, eculizumab	Alive	5
STAT3 GOF	7	Molecular diagnosis	Acute cutaneous GvHD	CMV reactivation; HHV6 reactivation: severe Norovirus enterocolitis	Sirolimus	Alive	6
APDS	14	Refractoriness and toxicity with sirolimus	/	/	Sirolimus	Alive	6
DADA2	62	Refractory disease	Acute cutaneous GvHD	CMV reactivation	Methotrexate, cyclosporine, etanercept, rituximab	Alive	4
fHLH-UNC13D	7	HLH	/	HLH reactivation	HLH 2004 protocol	Died ad day +6 for HLH reactivation	6
XLP-1	4.5	HLH	Acute cutaneous and hepatic GvHD Chronic cutaneous GvHD	/	HLH 2004 protocol	Alive	3
BENTA syndrome	6	HLH	/	CMV reactivation; persistent hypogammaglobulinemia	HLH 2004 protocol	Alive	5
RAG	7	Refractory AHA	/	AHA	Rituximab	Alive	7
DCLREC1 mutation	6	Molecular diagnosis	/	Persistent hypogammaglobulinemia; LPD	Azathioprine, Rituximab	Alive	6

BENTA: B-cell Expansion with NF-kappaB and T-cell Anergy; DADA2: deficiency of adenosine deaminase 2; GOF: gain of function; GvHD: graft-versus-host disease; fHLH: familial HLH; HLH: hemophagocytic lymphohistiocytosis; PRES: posterior reversible encephalopathy; VOD: veno-occlusive disease; XLP-1: X-linked lymphoproliferative disease type 1.

## 4 Discussion

H-ID represents a significant diagnostic and therapeutic challenge in patients with IEI, and its management remains heterogeneous in daily clinical practice. This study analyzed a large cohort of patients with IEI and H-ID to provide an overview of the efficacy and safety of the most commonly adopted therapeutic approaches. Although with the limitations derived from the retrospective nature of the study, the potential referral bias, the heterogeneity of the sample in terms of IEI diagnosis, phenotypic expression, and therapeutic management, and the absence of systematic longitudinal immunophenotypic or functional markers of treatment response, this study provides some relevant insights and highlights clinical and research perspectives, particularly regarding targeted therapies and phenotypic-directed approaches.

In H-ID, achieving a definitive diagnosis often requires a considerable time, and genetic analysis is not informative in a relevant percentage of patients (45). Therefore, as patients are rarely eligible for targeted therapies at H-ID onset, there is an urgent need for effective therapeutic approaches based on the clinical phenotype and suspected underlying IEI.

In this cohort, the choice of the therapeutic agents for H-ID was significantly influenced by the category of IEI (i.e. ALPID, humoral immunodeficiency) and the phenotypic expression (AIC or pLPD alone or combination of more clinical features). Patients with ALPID were most likely to receive sirolimus as a first-line agent, while MMF and rituximab were more often used as a first approach in patients with a diagnosis of humoral immunodeficiency or CID. Also, the wide spectrum of extra-hematological manifestations of immune dysregulation influenced therapy selection, with a preferential introduction of MMF and rituximab in patients with multiple autoimmune conditions. Concerning efficacy, the use of sirolimus in patients with ALPID resulted in a measurable response in all patients, with a considerably high CR rate. These findings are in line with previously published literature, which demonstrates that the efficacy of sirolimus in patients with AIC is greater when patients present an ALPID-related clinical phenotype (46–48). The molecular mechanisms underlying the efficacy of sirolimus are different and partly unexplored. In ALPS-FAS and ALPS-sFAS, where it can be considered a targeted therapy, sirolimus acts through the induction of apoptosis of double-negative T-cells and induction of regulatory T cells (49, 50). In other IEI-associated AIC, sirolimus can significantly revert the imbalance in T-cell subsets, such as the expansion of follicular helper T-cells and the T-helper 1 skewing, as recently demonstrated in a study by Kumar et al. (51, 52). Moreover, the pathogenic overlap between ALPS-FAS and other diseases of the ALPID spectrum provides the rationale for the efficacy of sirolimus even in these conditions (47).

Notably, most of the previous studies have been conducted on patients with AIC, independently from the association with pLPD. This represents a considerable bias in assessing the overall efficacy of medical treatment, since, even in the same patient, the clinical response can be restricted only to some of the dysregulation features.

In this cohort, it is noteworthy that patients treated for isolated pLPD were more likely to receive sirolimus. Nevertheless, a relevant proportion of patients with isolated pLPD did not receive specific treatment. This raises is an intriguing point for further discussion, since the indication to treat this category of patients is not univocally accepted. Indeed, while the risk of developing clonal LPD in this subset of patients is well-known (53, 54), the potential benefits of treating isolated pLPD with immunomodulating/immunosuppressive agents to prevent this evolution have to be more deeply explored. Likewise, the optimal duration of treatment in patients with pLPD is not clearly defined. Given that, in IEIs, some features of immune dysregulation can show a progressive attenuation with age (55), identifying an appropriate timing to discontinue therapy is also of clinical relevance.

Further studies on the treatment of pLPD are warranted, to identify potential predictors of clonal evolution and treatment need/response in isolated pLPD, and finally provide tailored strategies. Potential areas for investigation could include serum biomarkers, imaging features, and, hopefully, the analysis of the somatic mutational pattern in tissues involved in lymphoproliferation (56).

Some limitations to the adoption of LTT derive from concerns over the infectious risk in patients with IEI receiving immunomodulating agents. However, this study confirms that severe AEs are uncommon in patients with H-ID receiving long-term therapies, even after a considerable follow-up. Specifically, despite the underlying condition of immune impairment, the rate of severe infections is low for all the main therapeutic agents, although with a slight prevalence in those treated with sirolimus. This is in line with previously published studies (57, 58), including a recent paper by Comella et al., in which the overall rate of severe infection was below 15% (57). This can partly be explained by the fact that some drugs, and especially those used as targeted treatments, can act as “disease-modifying” agents, thus also contributing to reverting the underlying immune defect (51, 52, 59, 60). Despite this, no significant difference in the infectious rate was evidenced between patients undergoing targeted and non-targeted treatments in this study. This can be explained by the low number of patients receiving targeted treatments and also by the high number of previous therapeutic lines used in some patients of this subgroup.

Another relevant issue is the low response rate observed in patients receiving on-demand treatments and TPO-Ras. Indeed, only one-third of patients with AIC showed a complete response following treatment with IVIG or corticosteroids. This data is consistently lower when compared with literature about the response to on-demand treatments in ITP and AHA outside of the IEI setting (61–63). Similarly, although with the limitations deriving from the small number of treated patients, also the response to TPO-Ras was suboptimal in the majority of patients (64–67). These results, together with the frequent need for further therapeutic lines in our cohort, reinforce the concept that therapeutic refractoriness in H-ID should be considered as a potential warning sign for an underlying IEI and lead to prompt immunological assessment (36, 68, 69).

In this cohort, the proportion of patients receiving targeted therapies was low, regarding fewer than 20% of the patients undergoing LTT. The rate of molecular diagnosis is consistent with data reported in literature, thus representing a reliable “real-life” scenario in terms of patients potentially eligible for targeted therapies. Indeed, according to literature data, the detection rate for genetic variants associated with CVID is reported to be around 25-30%, while a definitive diagnosis is reached in 35-40% of the patients presenting with ALPID features (70, 71). Also, the frequency of ALPS-FAS and ALPS-sFAS in this study, as well as the other targetable monogenic diseases, is similar to previously reported data. The emergence of targeted therapies, together with their increasing availability, is leading to a considerable improvement in the management of patients with immune dysregulation. Literature data report high response rates for the most commonly adopted targeted treatment. Large cohort studies pointed out an overall response rate of about 90% after treatment with JAK inhibitors in patients with STAT1 GOF or STAT3 GOF (72) and a sustained response in about 80% of patients with CTLA-4 or LRBA haploinsufficiency treated with abatacept (73). Similarly, data on the use of leniolisib in APDS are strongly encouraging (58). Notably, most studies analyze the outcome of targeted therapies without providing a direct comparison with other therapeutic strategies.

Patients receiving targeted therapies in our cohort had a significantly higher rate of CR compared to patients undergoing non-targeted treatments. Although the sample size is limited and data have been collected retrospectively, this result reinforces the importance of reaching a molecular diagnosis in patients with H-ID, to allow the identification of those carrying a druggable genotype. Of note, the incidence of AEs was slightly higher in patients receiving targeted therapies. This can potentially result from the higher baseline disease severity in this subgroup of patients, as well as the considerable therapeutic load received, since targeted therapies have been mostly administered as further therapeutic lines.

Hopefully, advances in the knowledge of the genetic background of patients with IEI and H-ID will allow an increase in the rate of molecular diagnosis in patients presenting with H-ID, thus resulting in the wider adoption of targeted therapies and improved clinical outcome in this delicate category of patients. Concerning this, the increasing analysis of somatic mutations in patients with high clinical suspect, such as in ALPS, as well as the continuous definition of new monogenic entities, represent promising areas for improvement (74–76).

Beyond eligibility for targeted therapies, our data, together with the available literature, do not allow firm conclusions regarding the optimal timing and duration of these treatments, or their role as a bridge to HSCT in selected patients (77). Indeed, HSCT indications in patients with H-ID are in continuous evolution. In clinical practice, several factors should be considered, including the underlying IEI, treatment response or refractoriness, disease severity, clinical expression (HLH, progressive lymphoproliferation), risk of progression to malignancy, and patient’s perspective (78–80).

Recent studies performed on the use of sirolimus, JAK inhibitors, and abatacept as bridges towards HSCT showed promising results, although data on HSCT outcome (graft function, immune reconstitution, complications, graft-versus-host disease) are still limited (72, 73, 81).

According to recent publications, the overall survival (OS) of patients with CTLA-4 haploinsufficiency after HSCT is about 75%, with 60% of patients receiving previous treatment with abatacept (82); moreover, the OS of patients with STAT-related disorders previously receiving JAK inhibitors is 91% (72). On the other hand, data about the use of leniolisib as a bridge therapy are limited, and HSCT is commonly reserved for refractory APDS (24, 83–85).

Patients undergoing HSCT in this study had different underlying diseases and received several pre-HSCT therapeutic lines, thus not allowing us to draw specific conclusions. However, long-term outcome was favorable in most patients. This, together with literature data, suggests that advances in donor availability, HSCT platforms, and toxicity management could significantly lead to further optimization of HSCT even in this category of patients. Moreover, as the mortality rate of IEI-associated HLH is considerable, as also evidenced in this cohort, early identification of monogenic IEIs associated with HLH can allow timely transplantation and significantly improve the patient’s outcome.

## 5 Conclusion

The approach to H-ID is still considerably variable, often involving different medical subdisciplines, and standardized treatment strategies are warranted. Our data underscore the importance of screening patients with refractory AIC for an underlying IEI and also confirm the efficacy of sirolimus in patients with H-ID and ALPID phenotypes. Moreover, although with the mentioned methodological limitations, this study suggests a higher efficacy of targeted therapies for H-ID compared to other treatments, therefore highlighting the need for increasing the rate of molecular diagnosis among patients presenting with H-ID. Of note, the most relevant contribution of this study is descriptive, and further investigations are warranted to confirm and integrate our findings. The most intriguing research perspectives in this delicate field include the expanding knowledge of the genetic background of IEI with hematological involvement, the conduction of H-ID-specific prospective studies, and the search for new candidate-targeted therapies and biomarkers associated with treatment response.

## Data Availability

The raw data supporting the conclusions of this article will be made available by the authors, without undue reservation.
