# Supplementary material for: Treating hematologic immune dysregulation in inborn errors of immunity: a real-life multicenter study
Source: Front Immunol. 2026 Jul 27;17:1854044. doi: 10.3389/fimmu.2026.1854044 (PMC13453812; doi:10.3389/fimmu.2026.1854044)
Supplement: Supplementary file 1 [file Table1.docx]

**Supplementary material**

**Manuscript:** *Treating hematologic immune dysregulation in inborn errors of immunity: a real-life multicenter study*” by Giorgio Costagliola et al.

**Supplementary table 1 - Outcome measures**

|  | **CR** | **PR** | **NR** |
| --- | --- | --- | --- |
| Immune thrombocytopenia (ITP)^18^ | Platelet count stable >100.000/μl without disease flares | Platelet count between 30.000-100.000/μl (at least doubling baseline count) with a decreased incidence of disease flares | Platelet count stable <30.000/μl (without doubling of the baseline count)  or  Persistence of disease flares without a significant reduction in their frequency |
| Autoimmune hemolytic anemia (AHA)^19^ | Normalization of hemoglobin levels in the absence of transfusion-dependency without signs of active hemolysis and absence of disease flares | Improvement of hemoglobin levels without normalization and/or persistence of signs of hemolysis | Persistence of a significant reduction of hemoglobin levels with signs of hemolysis  and/or  Transfusion-dependency |
| Autoimmune neutropenia (AIN)^20^ | Increase of absolute neutrophil count (ANC) to >1000/μl | Improvement in ANC without reaching the target of 1000/μl | Absence of improvement of ANC |
| Polyclonal lymphoproliferative disease (pLPD) | Disappearance of lymphoproliferative features | Reduction of lymphoproliferative features (in terms of size and/or metabolic activity) | Persistence of lymphoproliferative features without appreciable reduction compared to pre-treatment assessment  and/or  Development of new features of LPD during treatment |
| HLH treatment^21-23^ | Normalization of all of the HLH symptoms and laboratory markers:  -No fever  -No organomegaly  -No cytopenia  -Negativity for HLH markers (normal fibrinogen, triglycerides, ferritin, CD25)  -No central nervous system involvement. | Improvement, without normalization, of the HLH symptoms and laboratory markers:  -No fever  -Improved organomegaly  -Improved cytopenia  -Improvement of HLH markers (increased fibrinogen, decreased triglycerides, ferritin, CD25)  -No disease progression | -Lack of clinical and laboratory improvement according to the CR and PR criteria  -Disease progression |
| HLH prevention^23^ | No episodes of HLH or HLH-like disease during treatment.  Negativity of all HLH markers | Positivity of less than 3 clinical or laboratory markers after the achievement of disease remission | HLH relapse during treatment (defined by the positivity of at least 3 clinical or laboratory markers after the achievement of disease remission) |
| Overall response (for patients with multiple features of H-ID) | Achievement of CR for all the features of H-ID | Achievement of PR for at least one of the features of H-ID, without progression or NR in the remaining features | NR for all the features of H-ID |

**References (from main text)**

[18] Rodeghiero F, Stasi R, Gernsheimer T, Michel M, Provan D, Arnold DM, et al. Standardization of terminology, definitions and outcome criteria in immune thrombocytopenic purpura of adults and children: report from an international working group. Blood. 2009;113:2386–2393. doi: 10.1182/blood-2008-07-162503

[19] Hill QA, Hill A, Berentsen S. Defining autoimmune hemolytic anemia: a systematic review of the terminology used for diagnosis and treatment. Blood Adv. 2019 Jun 25;3(12):1897-1906. doi: 10.1182/bloodadvances.2019000036

[20] Fioredda F, Spanoudakis M, Skokowa J, Tamary H, Farruggia P, Almeida A, et al. European guidelines on treatment and supportive measures in chronic neutropenias: A consensus between the European Hematology Association and the EuNet-INNOCHRON COST Action based on a systematic evidence review. Hemasphere. 2025 Apr 16;9(4):e70113. doi: 10.1002/hem3.70113

[21] Bergsten E, Horne A, Aricó M, Astigarraga I, Egeler RM, Filipovich AH, Ishii E, Janka G, Ladisch S, Lehmberg K, McClain KL, Minkov M, Montgomery S, Nanduri V, Rosso D, Henter JI. Confirmed efficacy of etoposide and dexamethasone in HLH treatment: long-term results of the cooperative HLH-2004 study. Blood. 2017 Dec 21;130(25):2728-2738. doi: 10.1182/blood-2017-06-788349

[22] Locatelli F, Jordan MB, Allen C, Cesaro S, Rizzari C, Rao A, et al. Emapalumab in Children with Primary Hemophagocytic Lymphohistiocytosis. N Engl J Med. 2020 May 7;382(19):1811-1822. doi: 10.1056/NEJMoa1911326

[23] Zhang Q, Zhao YZ, Ma HH, Wang D, Cui L, Li WJ, et al. A study of ruxolitinib response-based stratified treatment for pediatric hemophagocytic lymphohistiocytosis. Blood. 2022 Jun 16;139(24):3493-3504. doi: 10.1182/blood.2021014860

**Supplementary table 2: Detailed genetic diagnoses**

| **Disease** |  | **Gene** | **Genetic variant** |
| --- | --- | --- | --- |
| ALPID | APDS-1 (4): | *PIK3CD* (NM_005026.5) | c.1574 A>C  c.3061G>A (n.3) |
|  | APDS-2 (1) | *PIK3R1* (NM_181523.3) | c.1300-2 A>G |
|  | ALPS-FAS (6); | *FAS* (NM_000043.6) | c.471_474delGACA (n: 2)  c.796 A>G  c.901G>T (n: 2)  c.651+1 G>A |
|  | ALPS-sFAS (2), | *FAS* (NM_000043.6) | c.576_576delA  c.778 G>A |
|  | ALPS-CASP10 (1) | *CASP10* (NM_032977.4) | c.310 C>T |
|  | NFKB2 deficiency (1), | *NFKB2* (NM_001322934.2) | c.1061 C>T |
|  | CTLA4 haploinsufficiency (5), | *CTLA4* (NM_005214.5) | c.394 G>A (n: 2)  c.374delG  c.208 C>T  c.218 C>T |
|  | STAT3 GOF (2), | *STAT3* (NM_139276.3) | c.2144 C>T  c.1986 T>G |
|  | STAT1 GOF (1), | *STAT1* (NM_007315.4) | c.1985A>C |
|  | PRKCD deficiency (1) | *PRKCD* (NM_006254.4) | c.1352+1 G>A |
| Other immune dysregulation disorders | XLP-1 (2); | *SH2D1A* (NM_002351.5) | c.47 G>A  120-kb deletion containing exons 2–4 |
|  | XLP-2 (2); | *BIRC4* (NM_001167.4) | c.1188 A>T (2) |
|  | fHLH-UNC13D (2); | *UNC13D* (NM_199242.3) | c.847 A>G; c.1847 A>G (comp het) (n: 2) |
|  | APECED (1); | *AIRE* (NM_000383.4) | c.415 C>T; c.769 C>T (comp het) |
|  | BACH2 deficiency (1) | *BACH2* (NM_021813.4) | Not specified |
| CVID and other humoral immunodeficiencies | NFKB1 haploinsufficiency (3), | *NFKB1* (NM_003998.4) | c.1519 A>G  c.864 G>T  c.926 A>G |
|  | Heterozygous TACI deficiency (5); | *TNFRSF13B* (NM_012452.3) | c.310 T>C (n: 2)  c.431 C>A  c.310 T>C  c.260 T>A |
|  | IKAROS haploinsufficiency (1) | *IKZF1* (NM_006060.6) | c.1505 G>T |
|  | IRF2BP2 deficiency (2) | *IRF2BP2* (NM_182972.3) | c.352 C>T (n: 2) |
| Syndromic IEI | 22q11.2DS (10) |  |  |
| Combined immunodeficiencies | RAG1 deficiency (2) | *RAG1* (NM_000448.3) | c.2917 C>T; c.455 C>T (comp het)  c.2521 C>T; *RAG1* 5′ partial deletion (comp het) |
|  | DCLRE1C (ARTEMIS) deficiency (1) | *DCLRE1C* (NM_001033855.3) | c.207_209delGTT; c.541_542insG (comp het) |
|  | BENTA syndrome (1) | *CARD11* (NM_032415.7) | c.1071 C>A |
| Autoinflammatory disorders | DADA2 (2); | *ADA2* (NM_001282225.2) | c.144delG (hom) (n: 2) |
|  | NLRP12-associated disease (2) | *NLRP12* (NM_144687.4) | c.1250 A>T  c.3046 C>T |
| Others | BCL11B-related disorder (1) | *BCL11B* (NM_138576.4) | c.1944_1965del |
|  | Kabuki syndrome (3) | *KMT2D* (NM_003482.4) | c.16360 C>T  c.5212 G>T  Not specified |

**Supplementary Table 3: Other manifestations of immune dysregulation**

|  | **Atopic disease** | **Rheumatic diseases** | **Endocrinopathy** | **Gastrointestinal disease** |
| --- | --- | --- | --- | --- |
| N | 19 (16.4%) | 18 (15.5%) | 15 (12.9%) | 18 (15.5%) |
| Details | - Eczema (7) - Asthma (2) - Allergic rhinitis-conjunctivitis (5) - Chronic/recurrent urticaria (3) - Angioedema (1) - Food allergy (1) - Drug hypersensitivity reactions (3) | - Systemic lupus erythematosus (1) - Sjogren syndrome (1), - Vasculitis (6), - Recurrent aphthous lesions (2) - Myositis (2), - Psoriasis (5), - Membranous nephritis (3), - Juvenile idiopathic arthritis (1) - Recurrent fever (1) | - Thyroiditis (14) - Type 1 Diabetes (4) - Autoimmune adrenalitis (2) | - Chronic gastritis (2) - IBD (3) - Celiac disease (3) - Chronic diarrhea-unspecified (4) - Chronic colitis/ileitis (3) - Autoimmune hepatitis/cholangitis (5) - Esophagitis (1) - Pancreatitis (1) |

**Supplementary table 4: Therapies administered for non-hematological immune dysregulation**

| **Drug** | **N** | **Indication** | **IEI diagnosis** |
| --- | --- | --- | --- |
| Dupilumab | 1 | Cutaneous granulomatosis | CID |
| Omalizumab | 1 | Chronic urticaria | CTLA-4 haploinsufficiency |
| Anakinra | 1 | Recurrent fever | NLRP12-associated disease |
| Canakinumab | 1 | Recurrent fever | NLRP12-associated disease |
| Mesalazine | 1 | Enteropathy | APDS1 |
| Azathioprine | 2 | Enteropathy | CTLA-4 haploinsufficiency (1), APDS2 (1) |
| Hydroxychloroquine | 1 | Arthritis | APDS2 |
| Ustekinumab | 2 | Enteropathy (1), complex hyperinflammatory phenotype (1) | CVID (1); NLRP12-associated disease (1) |

**Supplementary table 5- Response to HLH treatment**

| **Diagnosis** | **Clinical features** | **Treatment 1** | **Treatment 2** | **Treatment 3** | **HSCT** | **Outcome** |
| --- | --- | --- | --- | --- | --- | --- |
| APECED | HLH with hemolytic uremic syndrome | High-dose corticosteroids+ Anakinra (CR) | / | / | / | Alive |
| XLP-1 | Organ HLH (orbital localization) | HLH2004 protocol (PR) | Anakinra (PR) | Rituximab (CR) | Yes | Alive |
| STAT-1 GOF | HLH | High-dose corticosteroids+ Anakinra (CR) | Ruxolitinib (CR-for relapse prevention) |  | / | Alive |
| fHLH-UNC13D | CNS HLH | HLH2004 protocol (PR) | / | / | Yes | Died for early HSCT complications |
| fHLH-UNC13D | HLH with pulmonary involvement | / | / | / | / | Died before starting HLH treatment |
| NLRP12-associated disease | HLH (4 episodes) | High-dose corticosteroids+ Anakinra (CR) | Canakinumab (PR) | Ruxolitinib (PR) | / | Alive |
| XLP-1 | EBV-associated HLH | HLH2004 protocol (NR) | Rituximab (NR) |  | / | Died before undergoing HSCT |
| XLP-2 | HLH | HLH2004 protocol (CR) | Rituximab (CR) | / | / | Alive |
| BENTA | HLH | HLH2004 protocol (CR) | Anakinra (CR) | / | Yes | Alive |
| APDS | HLH-like episode | Anakinra (CR) | / | / | / | Alive |
| ALPID | HLH | / | / | / | / | Alive |
